# Non-coplanar Arc-Involved Beam Arrangement With Sufficient Arc Rotations Is Suitable for Volumetric-Modulated Arc-Based Radiosurgery for Single Brain Metastasis

**DOI:** 10.7759/cureus.67265

**Published:** 2024-08-20

**Authors:** Kazuhiro Ohtakara, Kojiro Suzuki

**Affiliations:** 1 Department of Radiation Oncology, Kainan Hospital Aichi Prefectural Welfare Federation of Agricultural Cooperatives, Yatomi, JPN; 2 Department of Radiology, Aichi Medical University, Nagakute, JPN

**Keywords:** dose conformity, non-coplanar arc, stereotactic radiosurgery, volumetric-modulated arc therapy, brain metastasis

## Abstract

Introduction

In linac-based stereotactic radiosurgery (SRS) leveraging a multileaf collimator (MLC) for brain metastasis (BM), volumetric-modulated arcs (VMAs) enable the generation of a suitable dose distribution with efficient planning and delivery. However, the arc arrangement, including the number of arcs, allocation, and rotation ranges, varies substantially among devices and facilities. Some modalities allow coplanar arc(s) (CA(s)) or beam(s) alone, and some facilities only use them intentionally despite the availability of non-coplanar arcs (NCAs). The study was conducted to examine the significance of NCAs and the optimal arc rotation ranges in VMA-based SRS for a single BM.

Materials and methods

This was a planning study for the clinical scenario of a single BM, including 20 clinical cases with a gross tumor volume (GTV) of 0.72-44.30 cc. Three different arc arrangements were compared: 1) reciprocating double CA alone of each 360º rotation with different collimator angles of 0 and 90º, 2) one CA and two NCAs of each 120º rotation with the shortest beam path lengths to the irradiation isocenter (NCA_L), and 3) one CA of 360º rotation and two NCAs of each 180º rotation (NCA_F). The three arcs were allocated similarly to equally divide the cranial hemisphere with different collimator angles of 0, 45, and 90º. Three VMA-based SRS plans were generated for each GTV using a 5 mm leaf-width MLC with the identical optimization method that prioritized the steepness of dose gradient outside the GTV boundary without any constraints to the GTV internal dose. A prescribed dose was uniformly assigned to the GTV *D*_V-0.01 cc_, the minimum dose of GTV minus 0.01 cc. The GTV dose conformity, the steepness of dose gradients both outside and inside the GTV boundary, the degree of concentric lamellarity of the dose gradients, and the appropriateness of the dose attenuation margin outside the GTV boundary were evaluated using metrics appropriate for each.

Results

The arc arrangements including NCAs showed significantly steeper dose gradients both outside and inside the GTV boundary with smaller dose attenuation margins than the CAs alone, while NCAs showed no significant advantage on the GTV dose conformity. In the NCA-involved arc arrangements, the NCA_F was significantly superior to the NCA_L in terms of the GTV dose conformity, the steepness of dose gradient outside the GTV, the degree of concentric lamellarity of the dose gradients outside and inside the GTV boundary, and the appropriateness of dose attenuation margin. However, the NCA_F showed no significant advantage on the steepness of dose increase inside the GTV boundary over the NCA_L. The dose increase just inside the prescribed isodose surface to the GTV boundary was significantly steeper with the NCA_L than the NCA_F.

Conclusions

In VMA-based SRS for a single BM, an arc arrangement including NCAs is indispensable, and sufficient arc rotations are suitable for achieving a dose distribution that maximizes therapeutic efficacy and safety in comparison to limited ones which are appropriate for dynamic conformal arcs. Although VMA with CAs alone can provide a non-inferior GTV dose conformity to NCAs, CA(s) alone should be applied only to situations where shorter irradiation time is prioritized over efficacy and safety.

## Introduction

For brain metastases (BMs), localized treatment of the lesions in coordination with systemic therapy tends to be prioritized while preserving whole-brain radiotherapy, just as whole-organ irradiation is rarely applied to metastases to the lungs or liver [1]. Single- or multi-fraction stereotactic radiosurgery (SRS) has become the mainstream of local treatment due to its minimal invasiveness and high therapeutic efficacy [1], while the dose and distribution and resulting local treatment outcomes vary considerably among facilities [2,3]. Since the dawn of SRS, multiple non-coplanar beams or arcs have been adopted to achieve excellent dose concentration with suitable dose conformity and steep dose gradients at the target boundary. However, SRS is currently performed using a variety of devices and irradiation techniques, even with modalities that are only capable of coplanar arc(s) (CA(s)) [4].

Dynamic conformal arcs (DCAs) are one of the most common techniques for SRS using a linac equipped with a multileaf collimator (MLC) [2,5]. In the general forward planning, the dose heterogeneity of a target volume (TV) needs to be determined and assigned in advance, e.g. 70% isodose covering [2,5], and a contour-adjusted dummy structure (modified TV) for MLC adaptation frequently needs to be used to adjust and improve the dose conformity to the TV [6,7]. The arcs usually include at least two non-coplanar arcs (NCAs) and are allocated within the limited rotation ranges of around 120º to minimize the beam path lengths to the irradiation isocenter [2,5-8]. Meanwhile, some modalities limit the cephalad tilt (couch rotation) range of the NCA to <40º, compromising dose distribution [9]. 

In comparison to DCAs, volumetric-modulated arcs (VMAs) provide a superior dose distribution and efficient planning and delivery for SRS of BMs and are particularly effective for irregularly shaped and/or multiple lesions in close proximity [8,10]. Meanwhile, some facilities that can utilize VMA, including NCAs, perform SRS intentionally using only CA(s) [4,8]. VMA-based SRS with CA(s) alone definitely shortens the treatment time and may be less susceptible to intra-fractional cranial misalignment by omitting the couch rotation [11]. However, the omission of NCAs may compromise some of the suitable properties of dose distribution [8]. Thus, it is important to determine the optimal arc arrangement for effective and efficient treatment using VMA for a single lesion.

At our facility since 2018, a physical dose with the biologically effective dose (BED) of ≥80 Gy in a versatile and flexible dose fraction has been assigned to the gross tumor volume (GTV) boundary without lowering the prescribed dose even for large tumors localized in an eloquent area [10,12]. Furthermore, the GTV coverage objective has been increased from ≥98% to <0.01 cc of the uncovered volume (*D*_V-0.01 cc_) to further enhance the maximal tumor response and subsequent sustainability from the beginning of 2024 [13]. To maximize a GTV dose conformity and steepen the dose falloff outside the GTV boundary, an extremely inhomogeneous GTV dose with a steep dose increase inside the GTV boundary has been affirmatively accepted and is deemed as fundamentally preferable to enhance the anti-tumor efficacy [10,13,14]. In particular, a concentrically layered steep dose increase just inside a GTV boundary can lead to early tumor shrinkage and symptom alleviation during and after multi-fraction SRS [12,14-17]. However, larger tumors require an increase in the number of dose fractions of >5 and sufficient reduction of the surrounding brain dose to ensure long-term safety [12,18,19].

The study was conducted to examine the significance of NCAs and the optimal arc rotation ranges in VMA-based SRS for a single BM. Specifically, we clarified which elements of dose distributions based on CAs alone are disadvantageous compared to arc arrangements including NCAs and further examined whether limited arc rotations are optimal for VMA as well as DCA from multiple perspectives. 

## Materials and methods

This was a planning study for the clinical scenarios of a single BM and was approved by the Clinical Research Review Board of Kainan Hospital Aichi Prefectural Welfare Federation of Agricultural Cooperatives (20220727-1).

Twenty patients harboring BMs with various GTVs of 0.72-44.30 cc (median value: 11.41 cc; interquartile range (IQR): 4.81, 23.22 cc) were selected, and each original GTV was treated as a single lesion. The GTV was defined and contoured based on non-contrast-enhanced computed tomography (CT) images (voxel size 0.98 x 0.98 x 1 mm), T2-weighted images (WIs), and contrast-enhanced T1-WIs, using dedicated software MIM Maestro^®^ version 7.1.3 (MIM Software Inc., Cleveland, Ohio, USA), as in the previous study [14]. For in-depth dosimetric evaluation relevant to clinical outcomes, GTV + 2 mm, GTV - 2 mm, and GTV - 4 mm structures were generated by adding isotropic margins of 2 mm, -2 mm, and -4 mm, respectively, to each GTV boundary using MIM Maestro [14]. The case of GTV 0.72 cc was excluded from the GTV - 4 mm generation due to the small diameter.

**Figure 1:**
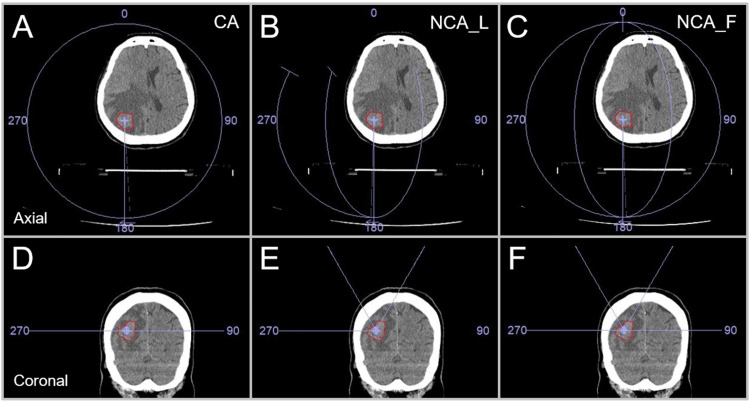
Three different arc arrangements compared. The images show head computed tomography (CT) images of a patient harboring a single brain metastasis (BM) in the right parietal lobe (A-F); the location of a gross tumor volume (GTV), the irradiation isocenter position, and three arc arrangement patterns (A, D; B, E; C, F); axial views (A-C); and coronal views (D-F). The three arc arrangements consist of two coplanar arcs (CAs) alone with to-and-fro rotations of 360º and each collimator angle of 0 and 90º (A, D); one coplanar arc (CA) and two non-coplanar arcs (NCAs) with each arc rotation of 120º to minimize the beam path lengths and the collimator angle setting of 0, 45, and 90º (B, E); and one CA with 360º rotation and the collimator angle of 0º and two NCAs with each 180º rotation and the collimator angles of 45 and 90º (C, F). The couch was rotated 60º clockwise and counterclockwise so that NCAs trisected the cephalad hemisphere (B, C, E, F). CA: coplanar arcs; NCA_L: non-coplanar arcs with limited rotations; NCA_F: non-coplanar arcs with full rotations.

In this study, a prescribed dose was assigned to the GTV *D*_V-0.01 cc_, the minimum dose to cover GTV minus 0.01 cc (*D*_>95%_ for GTV >0.20 cc or *D*_95%_ for GTV ≤0.20 cc) to ensure that the GTV below the prescribed dose is the minimum volume below a certain level, as described in the previous study [13]. The treatment platform was 5-mm MLC Agility^®^ (Elekta AB, Stockholm, Sweden) mounted in a linac Infinity^®^ (Elekta AB, Stockholm, Sweden) with a flattening filter-free mode of a 6 MV X-ray beam, which provides a dose rate of up to 1400 monitor unit per minute [10]. The planning system Monaco^®^ version 5.51.10 (Elekta AB, Stockholm, Sweden) was used to optimize VMA-based SRS plans [10,14]. Each irradiation isocenter was set at the GTV center. Three different arc arrangements with different collimator angle settings compared are shown in Figure 1.

In this study, the three arc arrangements were referred to as the CA (coplanar arcs), NCA_L (non-coplanar arcs with limited rotations), and NCA_F (non-coplanar arcs with full rotations), respectively.

The VMA plans were uniformly optimized using three cost functions in the Pareto mode with priority given to the steepness of dose falloff outside the GTV. The minimum volume (%) of the Target Penalty cost function was uniformly set to the same value for each GTV according to the corresponding coverage value of the GTV *D*_V-0.01 cc_ (98.61-99.98%) [14,20]. The other optimization settings and dose calculation algorithm were described in the previous study [14,20]. Following the completion of optimization, each GTV coverage by the prescribed dose was rescaled according to the corresponding coverage value of the GTV *D*_V-0.01 cc_ [14,20].

In this study, comparisons of the dose distributions using a conformity index (CI) or a gradient index were not intentionally performed [21,22]. These metrics are relative ratios and are influenced above all by the GTV itself [5,22]. These indices tend to show better values as the GTV increases [5,10,22]. Furthermore, even if the dose distribution is the same, these metrics are greatly influenced by the coverage value of a TV by the prescribed dose [21,22]. Insufficient GTV coverage by the prescribed dose likely improves the CI value itself [13,21]. Rather than these indices, the absolute doses and the isodose volumes irradiated to the surrounding normal tissue outside the GTV are likely more relevant to clinical outcomes [13,14,23-25].

An irradiated isodose volume (IIV) was defined as the volume irradiated with more than a certain relevant dose, including the GTV [23,24]. The IIVs of 100 (prescribed isodose volume, PIV), 75, and 50% of the GTV *D*_V-0.01 cc_ were calculated from the dose-volume histograms (DVHs). The volumes obtained by subtracting the GTV from these IIVs were then compared. The GTV near-maximum dose was evaluated with the *D*_0.01 cc_ for GTV ≥0.20 cc and *D*_5%_ (*D*_<0.01 cc_) for GTV <0.20 cc, instead of the *D*_2%_ or the maximum dose per voxel (0.001 cc unit) [14,26]. The GTV dose inhomogeneity was evaluated as the GTV *D*_V-0.01 cc_ (%) relative to the GTV *D*_0.01 cc_ (100%) [13]. The near-minimum doses of the GTV, GTV + 2 mm, GTV - 2 mm, and GTV - 4 mm were evaluated using the *D*_eIIV_ (eIIV: equivalent IIV), the minimum dose to cover the IIV equivalent to a reference TV on the DVH to avoid the substantial over- or under-coverage [14,20]. The coverage value of each TV by the *D*_eIIV_ reflects the degrees of dose conformity and the concentric lamellarity of dose gradients outside and inside the GTV boundary [14,20]. Evaluation of the coverage value of the GTV - 4 mm with the *D*_eIIV_ for the GTV of 1.26 cc was excluded due to the small volume of the GTV - 4 mm. The GTV dose conformity was compared using the smallness of the PIV spillage (cc) outside the GTV and the high GTV coverage value (%) of the GTV by the *D*_eIIV_ [14,20]. The steepness of dose gradients outside the GTV and GTV + 2 mm was compared using the smallness of the IIVs of 75 and 50% of the GTV *D*_V-0.01 cc_, excluding the GTV [10]. The appropriateness of a dose attenuation margin outside the GTV was compared using the GTV + 2 mm *D*_eIIV_ (%) relative to the GTV *D*_V-0.01 cc_ and the high coverage value of GTV + 2 mm by the *D*_eIIV_ [20]. The steepness of a dose increase inside the GTV boundary was compared using the *D*_eIIV_s (%) of GTV, GTV - 2 mm, and GTV - 4 mm, relative to the GTV *D*_V-0.01 cc_ (100%) [14]. In particular, the GTV *D*_eIIV_ reflects the steepness of dose increase just inside the prescribed isodose surface (IDS).

For statistical analyses, paired nonparametric tests were used, considering the distributions of the variables. Box-and-whisker plots (BWPs) were used to represent the distributions of variables. In the BWP, the whiskers denote the nearest values ≤1.5 times the IQR. The cross marks beyond the lines indicate the individual outliers >1.5 times the IQR. Friedman’s test (FT) and Scheffe’s post hoc test (SPHT) were used to compare three numerical variables. The Wilcoxon signed-rank test (WSRT) was applied to compare two numerical variables for which there was no significant difference in the SPHT despite a significant difference among three numerical variables in the FT. The comparisons consisted of CAs alone vs. a combination of a CA and NCAs and then limited (120º) vs. sufficient (180 and 360º) arc rotations. Therefore, the significant differences between the two groups in the WSRT were considered meaningful. Significance was considered at P < 0.05 (*), P < 0.01 (**) and P < 0.001 (***). Statistical analyses were performed using BellCurve for Excel (version 4.05; Social Survey Research Information Co., Ltd., Tokyo, Japan), in which the FT results may have been inaccurate in the previous version [10].

## Results

**Figure 2:**
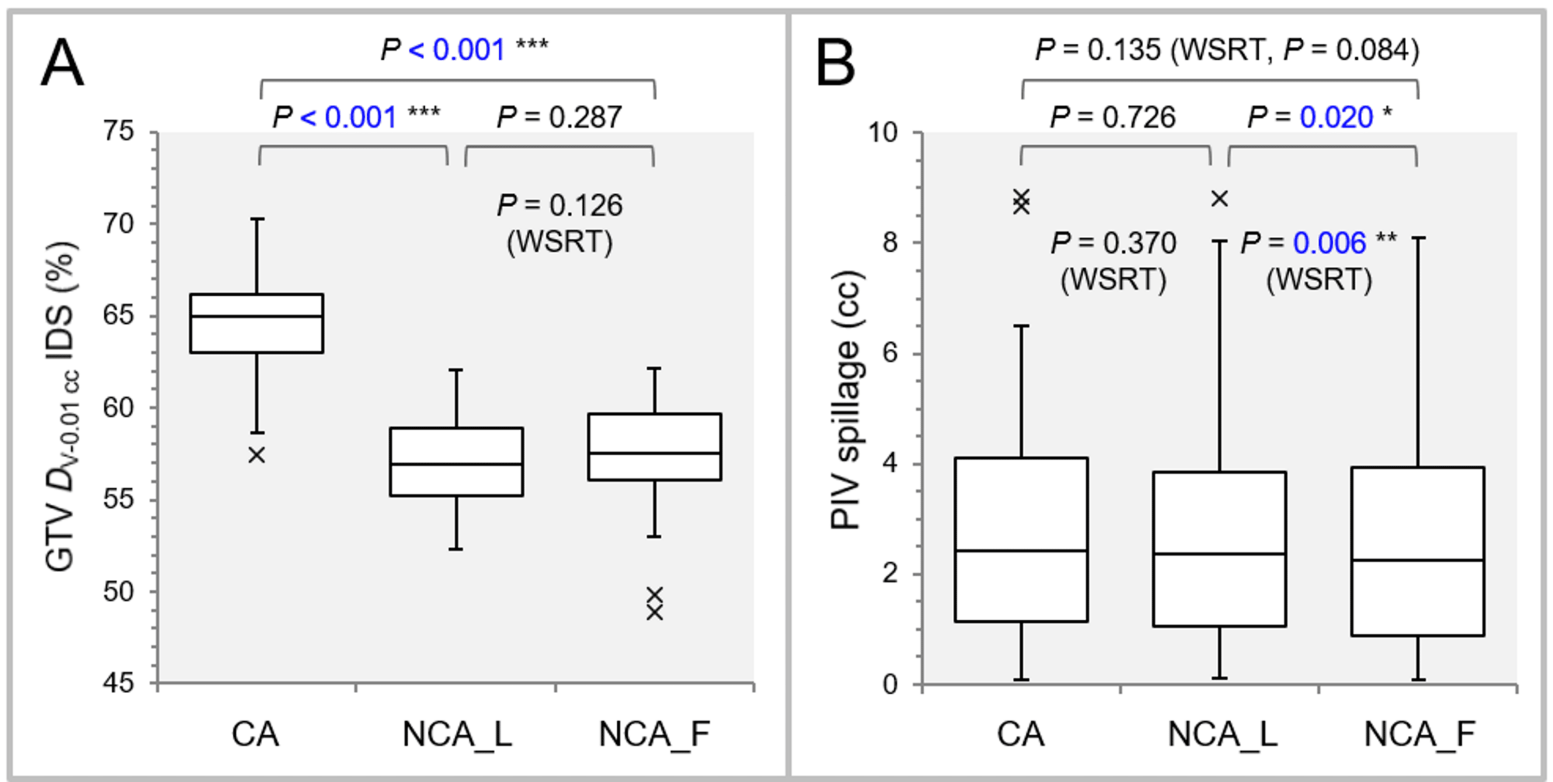
Comparisons of the GTV dose inhomogeneity and conformity. The images show box-and-whisker plots (BWPs) along with the results of Friedman’s test (FT), Scheffe’s post hoc test (SPHT), and Wilcoxon signed-rank test (WSRT) (A, B); the GTV *D*_V-0.01 cc_ (%) relative to the GTV *D*_0.01 cc_ (100%) to indicate the dose inhomogeneity (A); and the spillage volume (cc) of the irradiated isodose volume (IIV) of GTV *D*_V-0.01 cc_ outside the GTV to demonstrate the dose conformity (B). GTV: gross tumor volume; *D*_V-0.01 cc_: a minimum dose to cover a target volume (TV) minus 0.01 cc (*D*_>95%_ for TV >0.20 cc, *D*_95%_ for TV ≤0.20 cc); IDS: isodose surface; PIV: prescribed isodose volume; WSRT: Wilcoxon signed-rank test; CA: coplanar arcs; NCA_L: non-coplanar arcs with limited rotations; NCA_F: non-coplanar arcs with full rotations; *D*_0.01 cc_: a minimum dose covering 0.01 cc of a TV (*D*_0.01 cc_ for TV ≥0.20 cc and *D*_5%_ (*D*_<0.01 cc_) for TV <0.20 cc).

Figure 2 shows the comparative results of the GTV dose inhomogeneity and the PIV spillage outside the GTV.

FT demonstrated significant differences both in the GTV dose inhomogeneity (P < 0.001 ***) and the PIV spillage (P = 0.016 *) among the three groups. The GTV dose was significantly more inhomogeneous in the arc arrangements including NCAs (NCA_L, NCA_F) than the CAs alone. No significant difference in the GTV dose inhomogeneity was observed between the NCA_L and NCA_F, although the NCA_L tended to have more inhomogeneous GTV dose than the NCA_F in terms of the median value and IQR (Figure 1A). Regarding the PIV spillage outside the GTV, there was no significant difference between the CA and NCA_L or F, while the NCA_F had significantly smaller PIV spillage than the NCA_L (Figure 1B).

**Figure 3:**
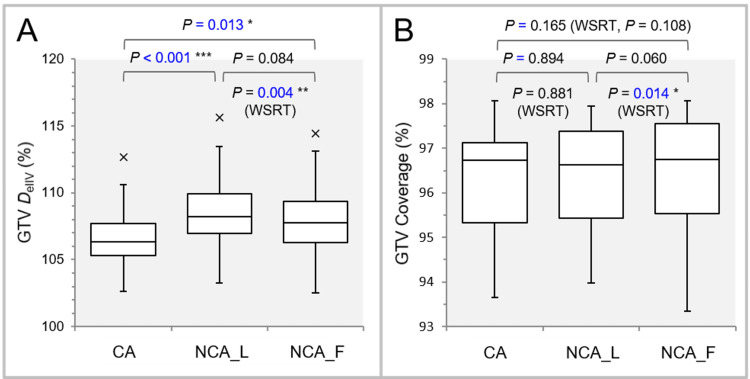
Comparisons of the GTV dose conformity by an alternative metric and the steepness of dose increase just inside the GTV DV-0.01 cc IDS. The images show BWPs along with the results of FT, SPHT, and WSRT (A, B); the GTV *D*_eIIV_ (%) relative to the GTV *D*_V-0.01 cc_ (100%) (A); and the GTV coverage value by the GTV *D*_eIIV_ to indicate the dose conformity alternatively (B). GTV: gross tumor volume; *D*_V-0.01 cc_: a minimum dose to cover a target volume (TV) minus 0.01 cc; IDS: isodose surface; *D*_eIIV_: the minimum dose to cover the irradiated isodose volume equivalent to a target volume (on the dose-volume histogram); WSRT: Wilcoxon signed-rank test; CA: coplanar arcs; NCA_L: non-coplanar arcs with limited rotations; NCA_F: non-coplanar arcs with full rotations; BWPs: box-and-whisker plots; FT: Friedman’s test; SPHT: Scheffe’s post hoc test.

Figure 3 shows the results of comparing the GTV *D*_eIIV_ and the coverage value.

FT showed significant differences in the GTV *D*_eIIV_ (P < 0.001 ***) and the coverage value (P = 0.043 *). The dose increase just inside the prescribed IDS (GTV *D*_V-0.01 cc_) was significantly steeper in the NCA_L and NCA_F than in the CA. The NCA_L had the highest GTV *D*_eIIV_, based on the results including the WSRT (Figure 3A). The NCA_F had significantly higher GTV coverage with the *D*_eIIV_ than the NCA_L, suggesting better dose conformity in the NCA_F, although the SPHT failed to reveal any significant difference (Figure 3B). 

**Figure 4:**
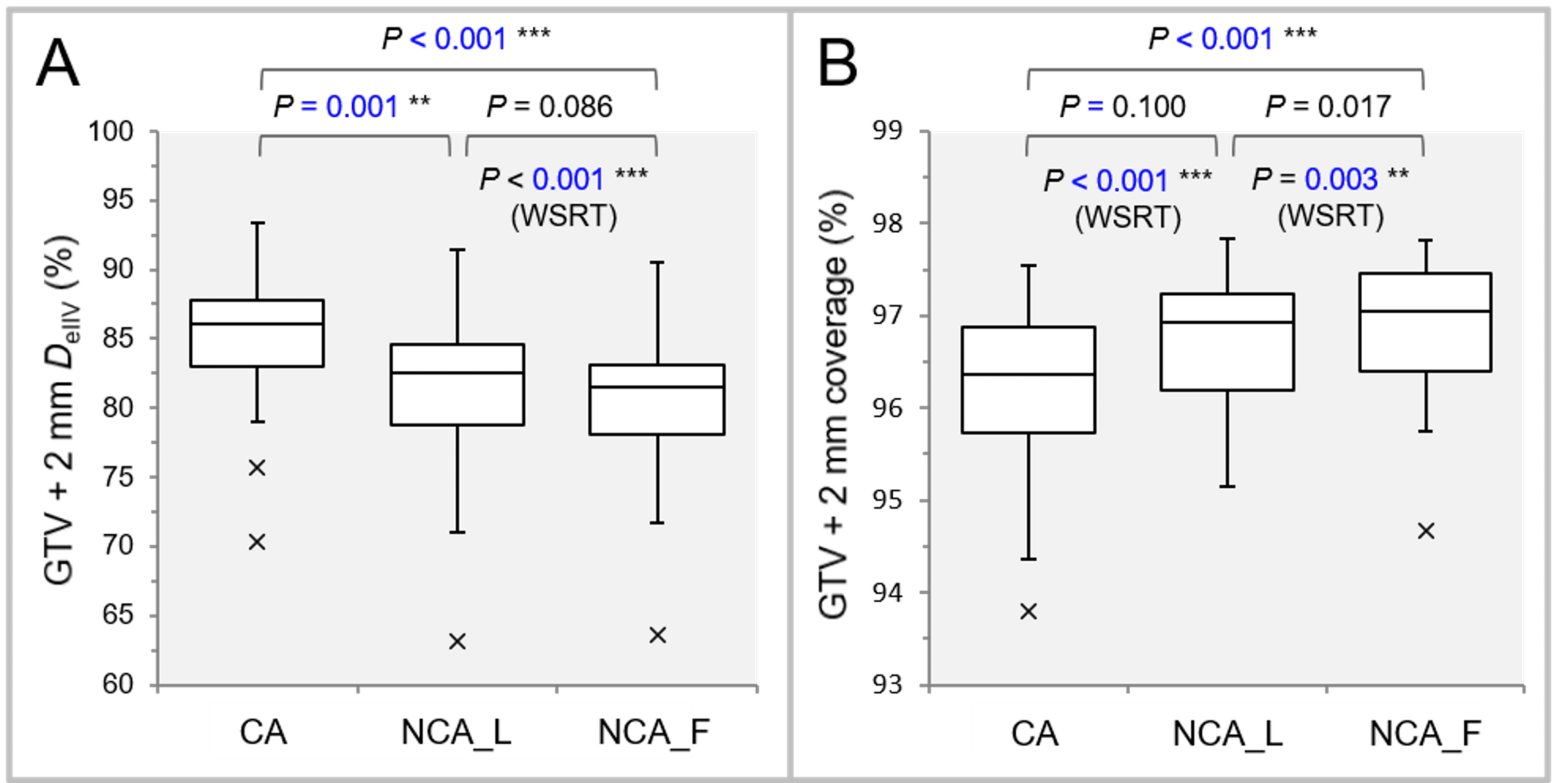
Comparison of the appropriateness of dose attenuation margin outside the GTV. The images show BWPs along with the results of FT, SPHT, and WSRT (A, B); *D*_eIIV_ (%) of the GTV + 2 mm relative to the GTV *D*_V-0.01 cc_ (100%) (A); and the coverage value of GTV + 2 mm by the *D*_eIIV_ to demonstrate the degree of concentric lamellarity of dose gradient outside the GTV boundary (B). GTV: gross tumor volume; GTV + 2 mm: GTV evenly expanded by 2 mm; *D*_eIIV_: a minimum dose to cover the irradiated isodose volume equivalent to a target volume; WSRT: Wilcoxon signed-rank test; CA: coplanar arcs; NCA_L: non-coplanar arcs with limited rotations; NCA_F: non-coplanar arcs with full rotations; BWPs: box-and-whisker plots; FT: Friedman’s test; SPHT: Scheffe’s post hoc test; *D*_V-0.01 cc_: a minimum dose to cover a target volume (TV) minus 0.01 cc; IDS: isodose surface.

Figure 4 shows the results of comparing the GTV + 2 mm *D*_eIIV_ and the coverage value.

FT demonstrated showed significant differences in the GTV + 2 mm *D*_eIIV_ (P < 0.001 ***) and the coverage values (P < 0.001 ***). The GTV + 2 mm *D*_eIIV_ was significantly lower in the NCA_L and NCA_F than in the CA. WSRT showed that the GTV + 2 mm *D*_eIIV_ was significantly lower in the NCA_F than in the NCA_L. There was one outlier with the GTV + 2 mm *D*_eIIV_ <65% to the GTV (0.72 cc) *D*_V-0.01 cc_ in the NCA_L and NCA_F, which were deemed excessively steep and needed to be adjusted [20]. WSRT demonstrated that the NCA_F had the highest coverage of the GTV + 2 mm by the *D*_eIIV_ among the three groups.

**Figure 5:**
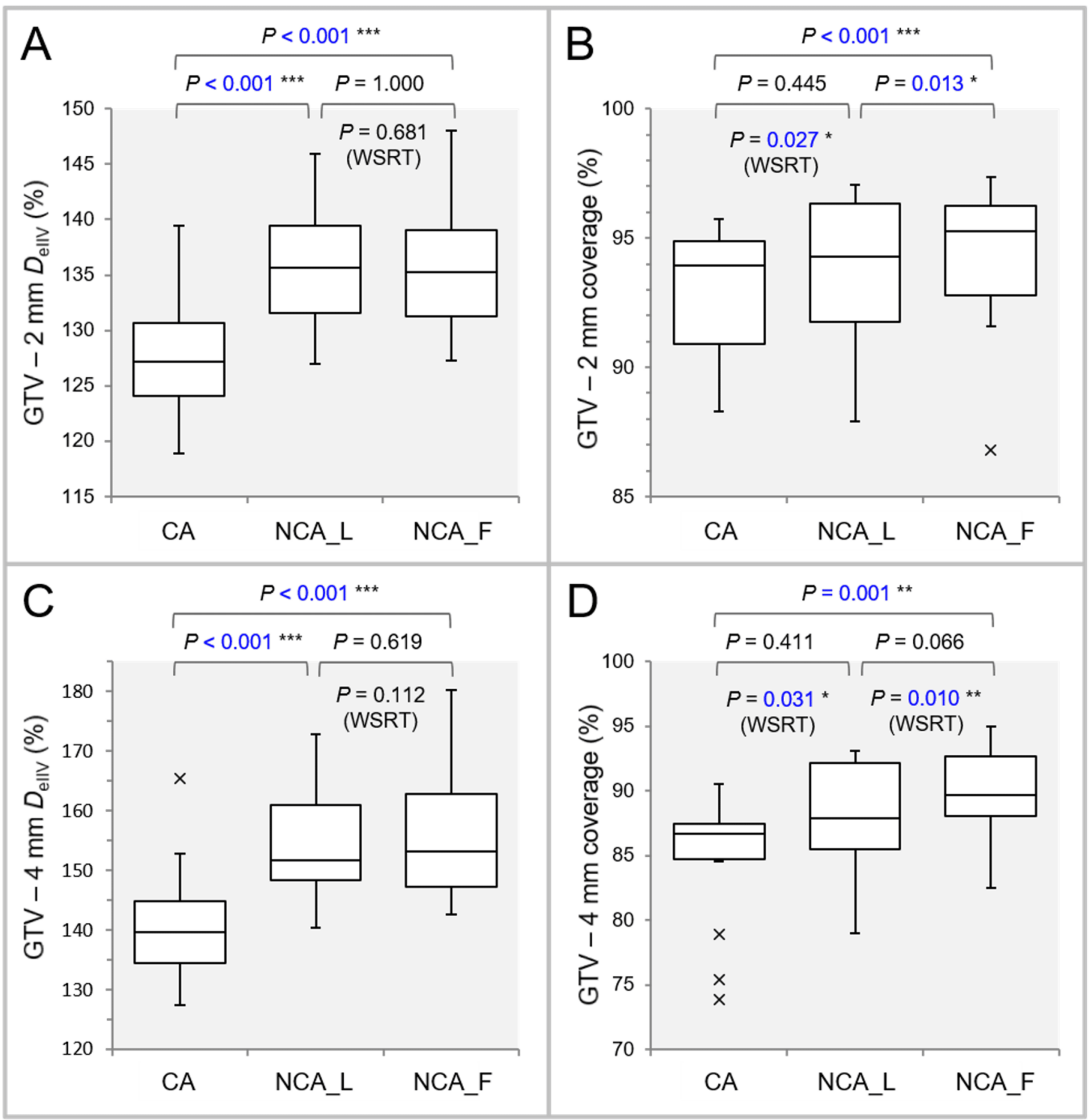
Comparison of the characteristics of the dose gradient inside the GTV boundary. The images show BWPs along with the results of FT, SPHT, and WSRT (A-D); *D*_eIIV_ (%) of the GTV - 2 mm (A) and GTV – 4 mm (C), relative to the GTV *D*_V-0.01 cc_ (100%), to indicate the steepness of dose increase inside the GTV boundary; and the coverage values of the GTV – 2 mm (B) and GTV – 4 mm (D) to demonstrate the degree of concentric lamellarity of dose increase. GTV: gross tumor volume; GTV - X mm: GTV evenly reduced by X mm; *D*_eIIV_: the minimum dose to cover the irradiated isodose volume equivalent to a target volume; WSRT: Wilcoxon signed-rank test; CA: coplanar arcs; NCA_L: non-coplanar arcs with limited rotations; NCA_F: non-coplanar arcs with full rotations; BWPs: box-and-whisker plots; FT: Friedman’s test; SPHT: Scheffe’s post hoc test; *D*_V-0.01 cc_: a minimum dose to cover a target volume minus 0.01 cc.

Figure 5 shows the results of comparing the *D*_eIIV_s of GTV - 2 mm and GTV - 4 mm and the coverage values.

FT showed significant differences in the GTV - 2 mm *D*_eIIV_ (P < 0.001 ***) with the coverage values (P < 0.001 ***) and the GTV - 4 mm *D*_eIIV_ (P < 0.001 ***) with the coverage values (P = 0.001 **). The *D*_eIIV_s of GTV - 2 mm and GTV - 4 mm were significantly higher in the NCA_L and NCA_F than in the CA, while WSRT also showed no significant difference between the NCA_L and NCA_F. FT and auxiliary WSRT showed that the NCA_F had the highest coverage values of both the GTV - 2 mm and GTV - 4 mm.

**Figure 6:**
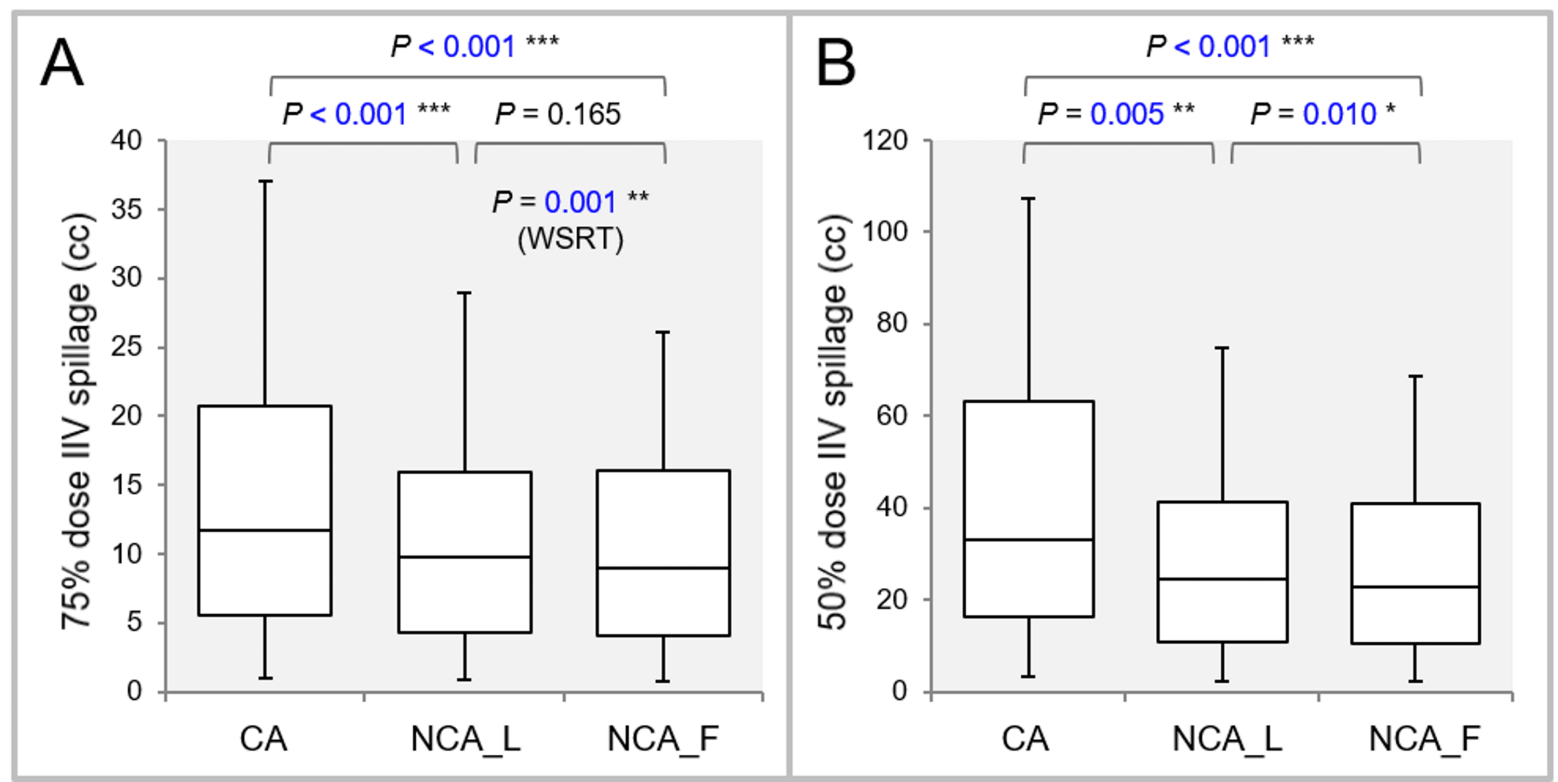
Comparison of the steepness of dose gradient outside the GTV boundary. The images show BWPs along with the results of FT, SPHT, and WSRT (A, B); and the spillage volumes (cc) of the IIVs with 75% (A) and 50% (B) of the GTV *D*_V-0.01 cc_ outside the GTV to demonstrate the steepness of dose gradient outside the GTV. GTV: gross tumor volume; IIV: irradiated isodose volume; WSRT: Wilcoxon signed-rank test; CA: coplanar arcs; NCA_L: non-coplanar arcs with limited rotations; NCA_F: non-coplanar arcs with full rotations; BWPs: box-and-whisker plots; FT: Friedman’s test; SPHT: Scheffe’s post hoc test; *D*_V-0.01 cc_: a minimum dose to cover a target volume (TV) minus 0.01 cc.

Figure 6 shows the results of comparing the spillage volumes irradiated with 75% and 50% of the GTV *D*_V-0.01 cc_, excluding the GTV.

FT demonstrated significant differences in the spillage volumes irradiated with 75% (P < 0.001 ***) and 50% (P < 0.001 ***) of the GTV *D*_V-0.01 cc_. Both the spillage volumes were significantly smaller in the NCA_L and NCA_F than in the CA. Furthermore, WSRT showed that both the spillage volumes were significantly smaller in the NCA_F than in the NCA_L.

**Figure 7:**
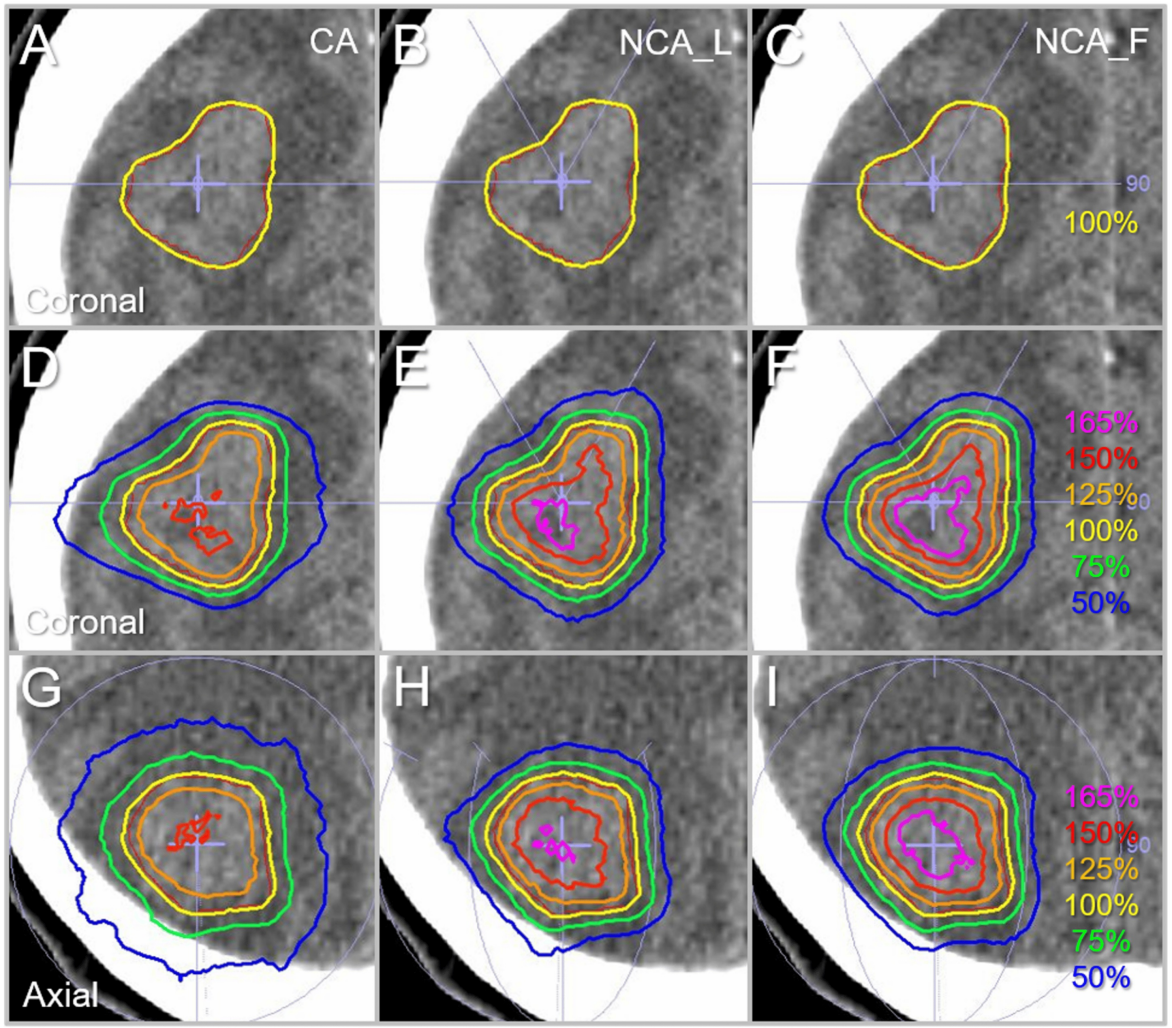
Comparison of the dose distributions for the GTV of 9.54 cc. The images show head CT images of a patient with a single BM (A-I), onto which the GTV contoured in red, arc arrangements, and representative isodoses are superimposed; coronal views with the most irregular GTV shape (A-F); and axial views (G-I). The isodose lines are shown as relative values with the GTV *D*_V-0.01 cc_ as 100% (yellow). GTV: gross tumor volume; CA: coplanar arcs; NCA_L: non-coplanar arcs with limited rotations; NCA_F: non-coplanar arcs with full rotations; CT: computed tomography; BM: brain metastasis; *D*_V-0.01 cc_: a minimum dose covering a target volume minus 0.01 cc.

Figure 7 shows the representative isodose distributions based on the three different arc arrangements for the GTV of 9.54 cc which was previously described in a case report [27].

There is no significant difference in the dose conformity of the GTV *D*_V-0.01 cc_ IDSs to the GTV boundary, while the dose gradients both outside and inside the GTV boundary are significantly different. The dose gradients are steepest with more pronounced concentric lamellarity in the NCA_F plan.

## Discussion

In the comparison of CAs alone vs. combination of a CA and NCAs, the latter arrangement failed to show superiority only in the GTV dose conformity, while being significantly superior in the other respects, regardless of the difference in the arc rotation ranges. The non-inferiority of the GTV dose conformity in the CAs alone is mainly attributed to the VMA optimization method without any dose constraints inside the GTV and the different collimator angle settings of 0 and 90º in the reciprocating double CA. Allowing high internal GTV doses without imposing dose constraints within the GTV generally leads to more suitable dose conformity to the GTV boundary [10,14]. In addition, although the central leaf width of the MLC examined was 5 mm, the orthogonal collimator angle combination enables the VMA optimization with the minimal segment size as small as 2.5 mm square, leading to improvement of the GTV dose conformity, in comparison to a single CA with a fixed collimator angle. Thus, although an excellent GTV dose conformality can be obtained with CA(s) alone through optimization using VMA, it should be noted that the dose gradients outside and inside the GTV boundary are significantly different from those of NCA and are generally unsuitable for SRS of BM(s). Therefore, CA(s) alone should be applied only to situations where shorter irradiation time is prioritized over efficacy and safety. In addition, a dose constraint within a GTV should be limited to situations where a homogeneous GTV dose and gradual dose precipitation outside the GTV are suitable, such as when the GTV contains some normal tissues whose functions should be preserved, and the tumor is highly invasive to the surrounding tissue [20].

In the arc arrangements including two NCAs, sufficient arc rotations with the CA of 360º and the NCAs of 180º provided superior overall dose distributions in comparison to all 120º rotations, in terms of dose conformity and gradient outside a GTV boundary, including the dose attenuation margin. In the limited arc rotations, the dose increase just inside the prescribed isodose surface to the GTV boundary was steeper than the sufficient rotations, however, there was no significant difference in the steepness of the dose increase further inside the GTV boundary. Furthermore, sufficient arc rotations lead to more concentrically layered dose gradients outside and inside a GTV boundary: superior dose conformity not only at the GTV boundary but also within several mm inside and outside the GTV boundary [14,20]. In an NCA-involved arc arrangement for VMA-based SRS, adequate arc rotations are recommended for single lesions.

In DCA with a constant dose rate and a gantry rotation speed, sufficient arc rotations of 180-360º likely lead to an unsuitable dose distribution with unnecessary dose spread around a TV. Therefore, an arc arrangement including at least two NCAs with limited arc rotations is common in DCA and is likely frequently applied similarly to VMA-based SRS for a single BM, while sufficient arc rotations are common when irradiating multiple lesions simultaneously with a single irradiation isocenter using either VMA or DCA with dedicated inverse planning [28]. It is noticeable that VMA with sufficient arc rotations, instead of limited rotations, can provide superior dose conformity and normal tissue sparing. When increasing dose fractionation and maintaining BED at the GTV margin for larger BMs, an arc arrangement including NCAs with sufficient rotations is preferable to minimize the surrounding tissue dose. Increasing the number of NCA to more than two may further improve dose distribution, while definitely prolonging the irradiation time and likely increasing intra-fractional errors. Therefore, a well-balanced arc arrangement to divide the cranial hemisphere into three equal parts, consisting of one CA and two NCAs, is generally suitable for MLC-based SRS of a single BM with either DCA or VMA [8].

Study limitations

This planning study includes inevitable inherent limitations, and whether an arc arrangement with sufficient arc rotations contributes to improved clinical outcomes remains unproven for single BMs, warranting further investigation. The superior dose distribution described above does not necessarily guarantee excellent clinical outcomes. Inferior dose conformity with a GTV over-coverage may compensate for unexpected uncertainties such as the GTV displacement during multi-fraction SRS or profound microscopic brain invasions [20]. In addition, a steep dose increase inside the GTV may lead to significant GTV shrinkage with gradual high-dose exposures to the surrounding brain during treatment, leading to the development of symptomatic brain radionecrosis [12]. Further studies are also warranted to determine whether similar results can be reproduced when using different MLCs and/or planning systems [29,30]. Although the arc rotation ranges were limited to the cranial hemisphere to secure sufficient distances between the head and the gantry in this study, the usefulness of arc rotation beyond that remains further investigation. Similar to the need for continuous training and research to improve surgical techniques in the realm of neurosurgery, planning studies in pursuit of more appropriate dose distribution should be continued to further enhance the efficacy and safety of SRS.

## Conclusions

In VMA-based SRS for a single BM, an arc arrangement including NCAs is indispensable, and sufficient arc rotations are suitable for achieving a dose distribution that maximizes therapeutic efficacy and safety, in comparison to limited ones which are appropriate for dynamic conformal arcs. Although VMAs with double CA alone with 360º rotation and different collimator angle settings of 0 and 90º can provide a non-inferior GTV dose conformity to NCA-included VMA, the dose gradients inside and outside the GTV boundary are significantly inferior in CA(s) alone. VMAs with CA(s) alone should be applied only to situations where shorter irradiation time is prioritized over efficacy and safety, especially for a large GTV.
